# Reduced Maximal Force during Acute Anterior Knee Pain Is Associated with Deficits in Voluntary Muscle Activation

**DOI:** 10.1371/journal.pone.0161487

**Published:** 2016-08-25

**Authors:** Sauro Salomoni, Kylie Tucker, François Hug, Megan McPhee, Paul Hodges

**Affiliations:** 1 The University of Queensland, Centre for Clinical Research Excellence in Spinal Pain, Injury and Health, School of Health and Rehabilitation Sciences, Brisbane, Australia; 2 The University of Queensland, School of Biomedical Sciences, Brisbane, Australia; 3 University of Nantes, Laboratory “Motricité, Interactions, Performance” (EA 4334), Nantes, France; Fondazione Santa Lucia Istituto di Ricovero e Cura a Carattere Scientifico, ITALY

## Abstract

Although maximal voluntary contraction (MVC) force is reduced during pain, studies using interpolated twitch show no consistent reduction of voluntary muscle drive. The present study aimed to test if the reduction in MVC force during acute experimental pain could be explained by increased activation of antagonist muscles, weak voluntary activation at baseline, or changes in force direction. Twenty-two healthy volunteers performed maximal voluntary isometric knee extensions before, during, and after the effects of hypertonic (pain) and isotonic (control) saline injections into the infrapatellar fat pad. The MVC force, voluntary activation, electromyographic (EMG) activity of agonist, antagonist, and auxiliary (hip) muscles, and pain cognition and anxiety scores were recorded. MVC force was 9.3% lower during pain than baseline (p < 0.001), but there was no systematic change in voluntary activation. Reduced MVC force during pain was variable between participants (SD: 14%), and was correlated with reduced voluntary activation (r = 0.90), baseline voluntary activation (r = − 0.62), and reduced EMG amplitude of agonist and antagonist muscles (all r > 0.52), but not with changes in force direction, pain or anxiety scores. Hence, reduced MVC force during acute pain was mainly explained by deficits in maximal voluntary drive.

## Introduction

Musculoskeletal pain is associated with reduced maximal voluntary contraction (MVC) force [1,2]. For example, maximal voluntary knee extension force is up to 60% lower in people with knee osteoarthritis [3–6] and anterior knee pain [7] than healthy controls. This reduction has been attributed to factors such as muscle atrophy [3,8], nociceptive-mediated central inhibition of the motor drive [3,4,7], as well as beliefs and cognition towards pain, such as fear and anxiety [1,9,10]. However, it is difficult to isolate the impact of pain on the generation of MVC force in these patients, as other factors could also be involved. When the effects of pain are investigated more specifically by painful injection of hypertonic saline, studies report smaller (< 20%) reductions in MVC force than those observed in patients with clinical pain: average reductions of 15–20% have been reported in the knee extensor [11,12], and 5% in the elbow flexor [13] muscles. Clarification of the mechanism(s) for reduced force during pain is important for understanding clinical conditions and has implications for the design of preventive/rehabilitative strategies.

As hypertonic saline does not affect contractile properties of muscle fibers [11,14], reduced force during pain must originate from reduced voluntary drive to the motoneurons, which can be estimated by the twitch interpolation technique [15]. The amplitude of the superimposed twitch response, elicited by electrical stimulation, increases when voluntary drive is reduced (i.e. submaximal), which may reflect reduced excitability of motoneurons and/or regions within the motor cortex. Although excitability of the motor pathways can be influenced by pain [1], previous investigations report no significant change in maximal voluntary activation during pain, despite reduced maximal force [13,16].

The failure to identify consistently reduced voluntary activation may be explained by several factors. First, MVC force may decrease because of greater activation of the antagonist muscles [13,16,17]. However, Henriksen et al. [12] reported reduced activation of both agonist and antagonist muscles in association with reduced maximal force of the knee during experimental knee pain. Second, the relationship between the level of voluntary drive to the motoneurons and the superimposed twitch response (interpreted as representing voluntary activation) has a non-linear sigmoidal shape [15]. Near maximal levels, a relatively large decrease in voluntary drive is required to produce a consistent increase in twitch response [18,19]. Hence, the failure to observe consistently reduced voluntary activation (i.e. reduced voluntary drive) in previous studies may have been caused by a predominance of individuals with high baseline levels of voluntary activation. In other words, individuals with high baseline levels of voluntary activation would show relatively small changes in twitch response during pain, which could be misinterpreted as small changes in voluntary drive. Third, experimental knee pain can result in changes in force direction [20]. Therefore, previous studies using single-axis force sensors may have misinterpreted changes in force direction during pain [20] as changes in force magnitude, which would affect the measure of MVC forces and twitch responses. Alternatively, Farina et al. [21] reported slower discharge rates and increased twitch force amplitude during pain. However, these changes were not correlated, suggesting the two mechanisms do not compensate when exerting a painful contraction.

Furthermore, attitudes and beliefs towards pain (e.g. fear and anxiety) contribute to reduced MVC force in patients with chronic pain [10,22], and may reduce the capacity to fully drive the motor pathway during an acute pain episode. Considering the potential effects in voluntary activation, these are mechanisms that warrant exploration. Although we considered it unlikely that participants with high fear of pain would volunteer, we expected that some variation in pain beliefs would be present between participants, which would enable consideration of this point.

Our objective was to directly assess whether these putative mechanisms could explain the reduction of MVC force during experimental pain, given the inconclusive evidence for reduced voluntary activation. We aimed to determine whether the reduction in maximal voluntary knee extension force during acute experimental pain can be explained by: (i) increased activity of antagonist muscles; (ii) individual differences in baseline levels of maximal voluntary activation; or (iii) modification of force direction. We also studied whether between-subject variation of MVC force during pain was related to psychological features associated with the pain experience. We hypothesized that experimental pain would reduce MVC force, and that this reduction would be associated with reduced voluntary activation of both agonist and antagonist muscles and would be larger in individuals with lesser baseline voluntary drive and/or unhealthier pain beliefs and/or higher anxiety.

## Methods

### Participants

Twenty-two volunteers (14 males and 8 females, age: 25 ± 5 years, weight: 71 ± 14 kg, height: 173 ± 11 cm, mean ± SD) with no history of knee pain participated in this study. Participants were recruited by public advertisement at the host institution. The study was approved by the Medical Research Ethics Committee at The University of Queensland, and all procedures conformed to the Declaration of Helsinki. All participants signed an informed consent prior to the experimental procedures.

### Experimental protocol

**Fig 1:**
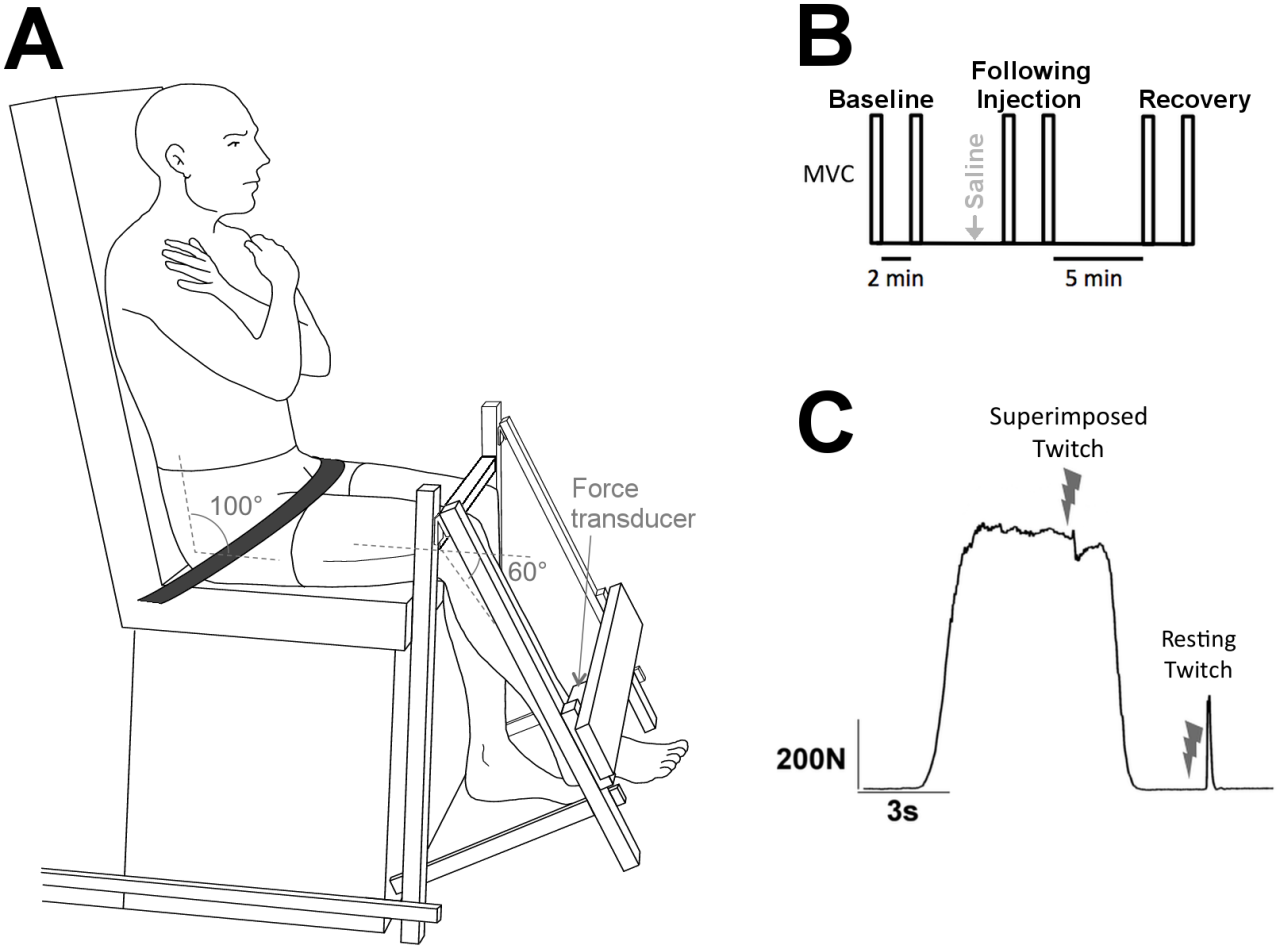
Experimental setup and protocol. **A**: Knee extension force was assessed with a three-dimensional sensor. Straps were placed on the hip, thighs and ankle (not shown) to limit body movements. **B**: Experimental protocol for each injection type. Participants performed two maximal voluntary contractions (MVC) at each time condition (baseline, following injection (< 3 min), recovery). **C**: Supramaximal electric stimulation was delivered to the quadriceps muscle during maximal voluntary contraction (MVC) and at rest.

Isometric knee extensions were performed with the dominant leg, determined by the preferred leg used to kick a ball. Participants sat in a custom designed chair with their back and thighs supported, and hip angle at 100° flexion (Fig 1A). The non-dominant leg hung freely at 90° knee flexion over the base of the chair, and the dominant leg was strapped at 60° knee flexion (0° = full knee extension). This knee angle has been associated with submaximal voluntary drive compared with 90° knee flexion [23]. Considering the sigmoidal relationship between the level of voluntary drive and the force response [15], this was considered an advantage for our study, as it minimizes potential non-linear ceiling effects at near-maximal voluntary activation. Closer to the linear range of the curve, changes in twitch force response are more likely to be detected, thus providing greater potential to study changes in voluntary activation during pain. The force sensor, rigidly attached to the base of the chair, was secured to the lower leg 5 cm proximal to the medial malleolus of the dominant leg. The height of a padded metal bar was adjusted to the distal part of the thigh and a support strap (~5 cm wide) was firmly secured proximal to the hip to minimize movement of the upper leg throughout the session.

A randomized, controlled crossover design was used, with each participant assessed in two separate sessions, separated by at least one week. At the beginning of each session, participants were familiarized with the setup by performing 3 isometric maximal voluntary contractions (MVC) of the knee extensors. Additional contractions were performed if the difference between consecutive trials was larger than 10%. For each MVC, participants increased their knee extension force over 1–2 s until they reached their maximum, and held this maximal effort for about 4 s. During familiarization, participants were provided with visual feedback. After familiarization, visual feedback was removed. Participants performed three sets of two MVCs: before, immediately following (< 3 min) injection of saline solution, and during recovery (Fig 1B). Supramaximal electrical stimuli were delivered during the hold phase and ~3 s after each MVC, evoking a superimposed twitch and a potentiated resting twitch, respectively (Fig 1C). Participants received strong verbal encouragement during each contraction. To minimize muscle fatigue, each MVC was separated by at least 2 min of rest, and a further 5 min rest was provided between conditions (i.e. baseline, following injection, and during recovery).

### Experimental knee pain

Acute anterior knee pain was induced by a single bolus injection of hypertonic saline (0.25 ml, 5.8%) into the medial part of the infrapatellar fat pad of the dominant leg. Isotonic saline (0.3 ml, 0.9%) was used as control. Injections were performed using a 1-ml plastic syringe with a disposable needle (25G, 25 mm). Pain intensity was assessed using a numeric rating scale (NRS) from 0 to 10, where 0 represents “no pain” and 10 represents “worst pain imaginable”. Participants rated the pain intensity immediately before each MVC, as a measure of pain at rest. Immediately following the second supramaximal electrical stimulation (i.e. when participants were again at rest), participants were asked to report the pain they had experienced during contraction. Participants understood that the rating referred to the pain elicited by the saline injection, not the electrical stimulation. Although some of the participants had received injections of saline in the past (8 out of 22), they reported pain and anxiety scores similar to those of naïve participants (both p > 0.7).

### Psychological assessment of pain and anxiety

In order to assess psychological factors that may influence pain adaptations, participants answered questionnaires related to their perception and beliefs about pain. These were: pain beliefs questionnaire [24], pain catastrophizing scale [25]; coping strategies questionnaire [26]; pain anxiety symptoms scale [27]; and state-trait anxiety inventory [28]. Questionnaires were sent electronically to participants after the second experimental session to avoid any potential influence of the questions on the participants’ pain cognition during the experimental procedures.

### Force and electromyography

Isometric knee extension force was assessed using a three-dimensional force sensor (Sensix, France). Myoelectrical activity was measured using surface electromyography (EMG) electrodes (Blue Sensor, Ambu, Denmark, Ag/AgCl sensor with 18/28 mm^2^ area and 22 mm of centre-to-centre spacing) in bipolar configuration over the vastus lateralis, vastus medialis and rectus femoris muscles of the dominant leg. In order to ensure that participants were not using the contralateral limb as a support to produce additional knee extension force, myoelectrical activity was also recorded bilaterally over biceps femoris (long head), gluteus maximus and erector spinae. Electrode placement followed standard recommendations from the SENIAM project [15,29]. The skin was cleaned using abrasive gel (Nuprep, D.O. Weaver & Co, USA) and alcohol, and a ground electrode was placed on the wrist. EMG data were pre-amplified (x 10), band-pass filtered between 10 and 1,000 Hz (Neurolog, Digitimer, UK), and sampled with force data at 2,000 Hz using a Power1401 Data Acquisition System with Spike2 software (Cambridge Electronic Design, UK).

### Electrical stimulation

A doublet electrical stimulus (400 V amplitude, 100 μs pulse duration, 10 ms inter-pulse interval) was delivered by a Digitimer DS7AH constant current stimulator (Digitimer, UK) with large surface electrodes (10 cm x 5 cm, 3M Health Care, USA) placed 5 cm below the inguinal crease (proximal electrode) and 5 cm above the superior border of the patella (distal electrode). The resting twitch of maximal amplitude was determined by applying stimulus of increasing intensity in steps of 10 mA every ~10 s, until knee extension force plateaued despite an increase in current intensity. To ensure maximal response throughout testing, supramaximal stimulus intensity was used, corresponding to 120% of the intensity that evoked a maximal resting twitch response (range: 144–264 mA).

### Data analysis

Force and EMG signals were assessed offline using MatLab software (The MathWorks, USA). The MVC force was calculated from the resultant force vector (Fx: medial-lateral, Fy: proximal-distal, Fz: extension) as the average force over 100 ms before the electrical stimulus (superimposed twitch), similar to previous studies [13,16]. Average force angles in the sagittal and coronal planes were calculated as atan(Fx/Fz) and atan(Fy/Fz), respectively. The EMG signals were digitally band-pass filtered between 20 and 400 Hz. The root mean square (RMS) of each EMG signal was calculated over a time window of 250 ms, from 300 ms to 50 ms before the stimulus, which avoided stimulus artifacts. The ratio between the RMS EMG of agonist (average of quadriceps muscles) and antagonist (biceps femoris) muscles was calculated to assess potential changes in co-contraction between knee extensor and flexor muscles. Force and EMG parameters were expressed as a percentage of the highest baseline value (pre-injection) to account for differences in strength and EMG amplitude between participants. The percentage of voluntary activation was estimated as 100×[1—(superimposed twitch/resting twitch)] [13,30].

### Statistical analysis

Force and EMG parameters were assessed using a two-way repeated-measures ANOVA (RM-ANOVA) with Condition (hypertonic, isotonic) and Time (baseline, following injection, recovery) as within-subject factors. Pain scores were also assessed using a two-way RM-ANOVA, with Condition and Muscle state (during contraction, at rest) as within-subject factors. Post hoc tests were performed using the Student-Newman-Keuls correction (SNK; corrected p-values are reported). The Pearson coefficient was used to assess if changes in MVC force (relative to baseline) were correlated with changes in voluntary activation, EMG amplitude, questionnaires, or changes in individual directional force component (Fx, Fy, Fz), as well as changes in force angle. The Pearson coefficient was also used to assess if greater reductions in MVC force were associated with raw baseline levels of voluntary activation (without normalization). Data are presented as mean ± SD throughout the text and mean ± SEM in the figures. Cohen’s d values were calculated as measures of effect size for the main outcomes (the standard deviation following hypertonic saline injection was used as the standardizer). Finally, to determine whether gender differences in pain perception may have affected the results [31], t-tests were performed on each parameter comparing male and female participants.

## Results

### Experimental knee pain

On average, injections of hypertonic saline elicited more pain than isotonic saline (3.7 ± 2.4 vs. 0.4 ± 0.8; main effect of Condition, F_1,21_ = 91.5, p < 0.001; Condition × Muscle state interaction, F_1,21_ = 38.6, p < 0.001). This difference was observed both during contractions (2.6 ± 2.2 vs. 0.3 ± 0.7, SNK p < 0.001) and at rest (5.1 ± 1.6 vs. 0.5 ± 0.7, SNK p < 0.001). Following injection of hypertonic saline, pain scores were lower during contractions than at rest (SNK p < 0.001), which was not observed with the isotonic injection (SNK p = 0.55). Pain scores reported after hypertonic saline were moderately correlated with anxiety scores (r = 0.48; p = 0.03), the CSQ score (r = 0.44; p = 0.04), and the STAI Y-1 score (r = 0.46; p = 0.03). Anxiety scores were not different between injection types (hypertonic: 2.3 ± 2.2; isotonic: 1.3 ± 1.7). No gender differences were observed in pain or anxiety scores (p > 0.29).

### MVC force and voluntary muscle activation

**Fig 2:**
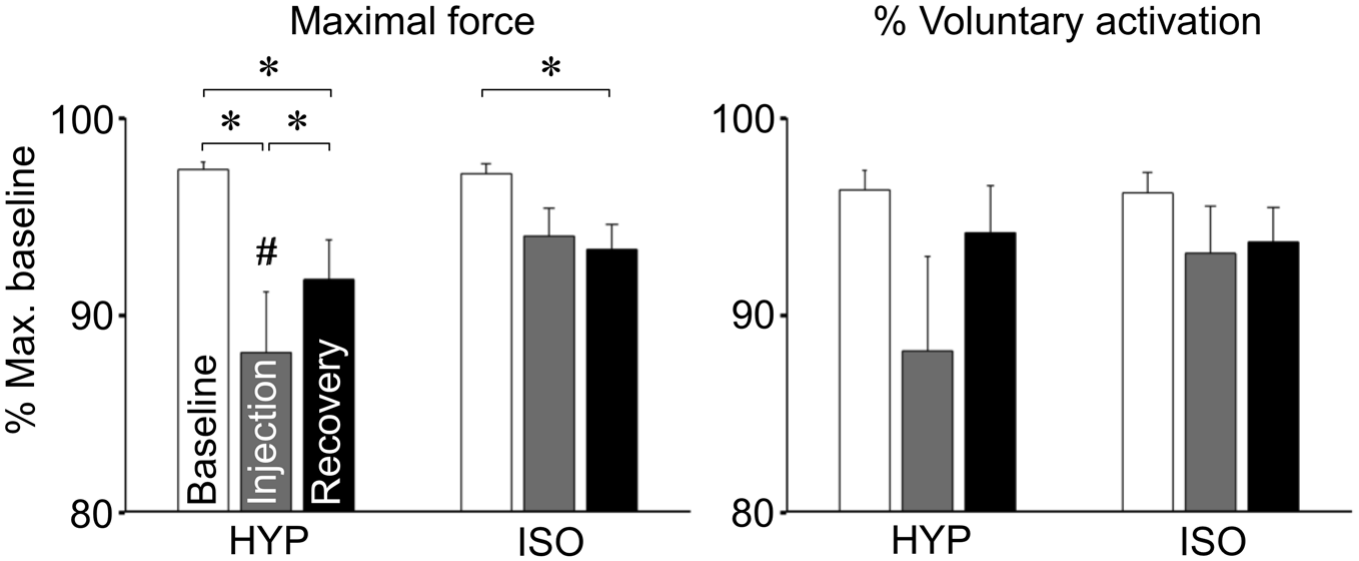
Maximal force and voluntary activation. Mean (SEM) maximal knee extension force and % voluntary activation of the quadriceps muscle before, immediately following painful (hypertonic) and control (isotonic) injections, and during recovery. * p < 0.05; # p < 0.05 vs. control.

The average MVC force across all participants was 636 ± 205 N (range: 287–1017 N) during baseline trials. Normalized total MVC force was lower following hypertonic saline than during baseline (Fig 2; 88.1 ± 14.4% vs. 97.4 ± 1.7%, Condition × Time interaction, F_2,42_ = 3.29, p = 0.047, SNK p < 0.001, Cohen’s d: 0.67) and following isotonic saline (94.0 ± 6.6%, SNK p = 0.008, Cohen’s d: 0.44), and increased again at recovery (91.8 ± 9.4%, SNK p = 0.04). The MVC force during recovery was lower than baseline for both hypertonic (91.8 ± 9.4% vs. 97.4 ± 1.7%, SNK p = 0.02, Cohen’s d: 0.40) and isotonic (93.3 ± 5.9% vs. 97.2 ± 2.3%, SNK p = 0.03, Cohen’s d: 0.28) injections, but not significantly different between injection types (SNK p = 0.39). Although significant, the reduction in MVC force following hypertonic saline was not systematically observed: only 11 out of 22 participants reduced MVC force by more than 5%.

**Fig 3:**
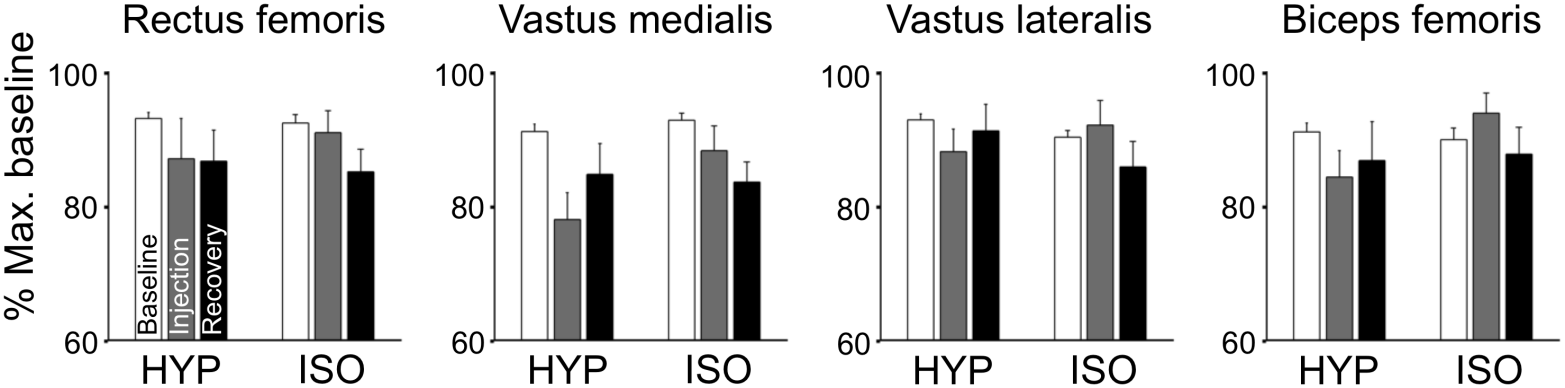
RMS EMG of agonist and antagonist muscles. Mean (SEM) RMS EMG of agonist (rectus femoris, vastus medialis, vastus lateralis) and antagonist (biceps femoris) muscles of the dominant leg. Contractions were performed before, immediately following painful (hypertonic) and control (isotonic) injections, and during recovery. No significant differences were found between conditions.

**Fig 4:**
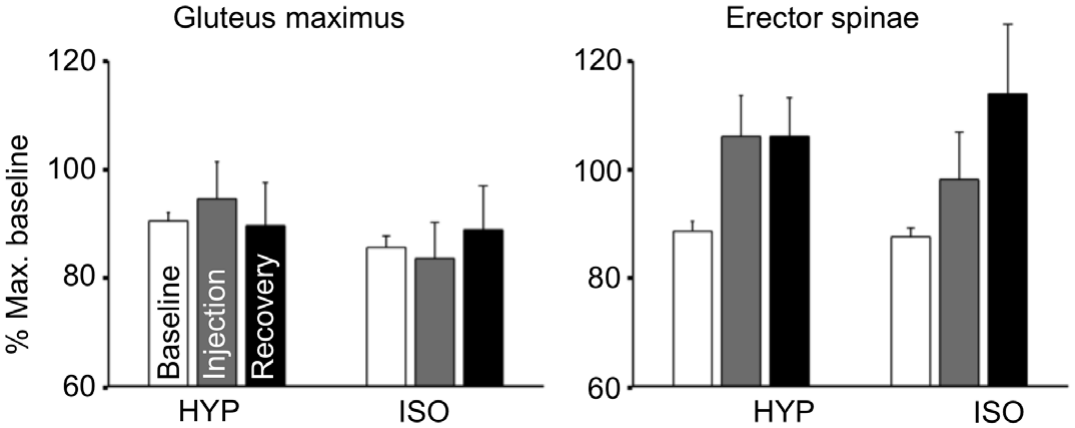
RMS EMG of auxiliary muscles. Mean (SEM) RMS EMG of auxiliary muscles (gluteus maximus, erector spinae) of the dominant leg during knee extensions performed before, immediately following painful (hypertonic) and control (isotonic) injections, and during recovery. No significant differences were found between conditions.

There were no significant changes in voluntary activation (Fig 2; Condition × Time interaction: F_2,42_ = 1.87, p = 0.17, Cohen’s d: 0.42 for hypertonic saline, 0.16 for isotonic saline). Similar to the MVC force, a large variability between participants was also observed: The average of non-normalized voluntary activation during baseline was 76 ± 18% (range: 27–97%). Also, no changes were found in RMS EMG of agonist muscles (vastus lateralis, vastus medialis and rectus femoris), antagonist muscle (biceps femoris), auxiliary muscles (gluteus maximus and erector spinae), or the muscles of the contralateral limb (biceps femoris, gluteus maximus and erector spinae) (Figs 3 and 4; Condition × Time interaction: all F_2,42_ < 2.32, all p > 0.1). Similarly, the agonist/antagonist ratio of RMS EMG did not change following hypertonic saline (Condition × Time interaction: F_2,42_ = 1.14, p = 0.3). No gender differences were observed in MVC force, voluntary activation, or EMG activity (p > 0.25).

The mean force angle did not change between conditions (mean angle: 8.7 ± 2.9 degrees lateral and 4.6 ± 5.4 degrees proximal from pure knee extension; p > 0.05). The amplitude of the resting twitch force response was higher during baseline than following injection and recovery (main effect of Time, F_2,42_ = 13.13, p < 0.001; SNK p < 0.001).

### Correlation analyses

Correlation analyses revealed associations between the reduction in MVC force following injection of hypertonic saline (relative to baseline) and other parameters assessed (Table 1). Despite the absence of systematic changes across individuals in voluntary activation and RMS EMG in the ANOVA, the reduction in MVC force was strongly correlated with changes in voluntary activation (r = 0.90, p < 0.001), and moderately correlated with changes in RMS EMG of agonist (rectus femoris r = 0.58; vastus medialis r = 0.78; vastus lateralis r = 0.57, all p < 0.006) and antagonist muscle (biceps femoris r = 0.52, p = 0.01).

**Table 1:** Correlation between changes in MVC force following injection of hypertonic saline and other parameters.

Parameter	r	p
Voluntary activation after hypertonic saline (pain)	**0.89**	**< 0.01**
Voluntary activation at baseline (not normalized)	**-0.62**	**< 0.01**
RMS RF	**0.58**	**< 0.01**
RMS VM	**0.78**	**< 0.01**
RMS VL	**0.57**	**< 0.01**
RMS BF	**0.52**	**0.01**
RMS GM	< 0.01	0.99
RMS ES	0.07	0.77
Pain	0.30	0.17
Anxiety	0.16	0.49
MVC Fx (medial-lateral)	**0.52**	**0.01**
MVC Fy (proximal-distal)	0.06	0.8
MVC Fz (knee extension)	**0.99**	**< 0.01**
Medial-lateral force angle	0.13	0.57
Proximal-distal force angle	-0.08	0.71

RMS—root mean square; RF—rectus femoris; VM—vastus medialis; VL—vastus lateralis; BF—biceps femoris; GM—gluteus maximus; ES—erector spinae; MVC—maximal voluntary force; Fx—medial-lateral force; Fy—proximal-distal force; Fz—extension force.

In contrast, the reduction in MVC force was *not* correlated with pain or anxiety scores (all r < 0.39, all p > 0.05) or with any of the pain cognition questionnaires (all r < 0.29; all p > 0.27). Baseline voluntary activation was *negatively* correlated with a reduction in MVC force (r = **−** 0.62; p = 0.002) and changes in voluntary activation (r = **−** 0.50; p < 0.02).

Changes in MVC total force were positively correlated with changes in knee extension force (Fz r = 0.99, p < 0.01) and medial-lateral force (Fx r = 0.52, p = 0.01) components. However, changes in force magnitude following hypertonic injection were not correlated with changes in force angle (medial-lateral r = 0.13, p = 0.57; proximal-distal r = -0.08, p = 0.71).

## Discussion

The present data supports that maximal isometric knee extension force is reduced during experimental knee pain in some, but not all, participants. The amount of force reduction following injection of hypertonic saline was strongly correlated with reduced voluntary activation, and moderately correlated with baseline levels of voluntary activation. The reduction in MVC force also correlated with reduced activity of both agonist and antagonist muscles. These findings suggest that individual differences in performance during pain are best explained by deficits in the voluntary drive. Changes in total force were associated with changes in the magnitude of both the knee extension and medial-lateral directional components, but not with changes in force angle, suggesting no differences in load sharing between muscles during painful maximal contractions. Taken together, these data highlight that individual variation of baseline voluntary activation, combined with the non-linear relationship between strength of the voluntary drive and twitch force response, provide the most likely explanation for the non-significant effect of pain on voluntary activation reported here and in previous studies. Pain and anxiety scores were not correlated with change in MVC force.

### Antagonist muscle activity

Increased activity of antagonist muscles has been proposed as a potential mechanism to reduce MVC force during acute pain [13,16,17]. This hypothesis is based on the proposal that group III and IV nociceptive afferents converge with group Ib (Golgi tendon organs) and group II (muscle spindles) afferents onto common interneurons, which may inhibit agonist and facilitate antagonist motoneurons during pain [17,32]. On the contrary, we found that reduced MVC force was positively correlated with reduced RMS EMG of both the agonist (quadriceps) and antagonist (hamstrings) muscles, with no changes in the agonist/antagonist ratio of RMS EMG during pain. Although reduced force was not systematically observed, participants with reduced MVC force also presented with a generalized reduction in the voluntary drive to agonist, synergist, and antagonist muscles, in agreement with previous observations [12,33].

### Deficits in voluntary activation

In the current study, acute anterior knee pain caused a small (~10 ± 15%) but significant reduction in maximal knee extension force. This is of the same order of magnitude as reported previously for the knee extensor [11,12,34] and elbow flexor [13] muscles during experimental pain.

Acute pain does not alter the membrane properties of muscle fibers, as indirectly confirmed by no change in the amplitude of the M-wave or the conduction velocity of action potentials [11,14]. Hence, reduced MVC force is most likely mediated by central mechanisms, i.e. reduced facilitation and/or inhibition, as indicated by the strong correlation between the reduction in MVC force and changes in voluntary activation. During acute experimental pain, the magnitude of motor-evoked potentials (MEPs) following transcranial magnetic stimulation (TMS) is smaller than in pain-free conditions [35–38], indicating reduced excitability of the corticomotor pathway. The lower MEP amplitude may be caused by reduced excitability at the cortical and/or spinal levels, but studies assessing biceps brachii EMG responses to both corticospinal and motor cortical stimulation suggest facilitation of motoneuron excitability and inhibition of motor cortical output during pain [35]. These findings suggest that the observed reduction in MVC force during pain is best explained by a reduction in the descending drive from the cortex rather than reduced motoneuron excitability. Mechanisms could include reduced intracortical facilitation and increased short-interval intracortical inhibition [38] or changes upstream of the motor cortex such as input from premotor areas.

Despite the significant reduction in MVC force during pain, the magnitude of change was not systematic across all individuals; some participants exhibited large changes in MVC force, whereas others showed little or no change. This variability was partially explained (r = 62) by differences in baseline levels of voluntary activation, i.e. participants with lower baseline voluntary activation showed greater reductions in MVC force following hypertonic saline. The sigmoidal relationship between the level of drive to the motoneurons and force magnitude results in a ceiling effect as the descending drive approaches the upper-range plateau. Similar reductions in voluntary drive would cause greater reduction in the superimposed twitch response when the voluntary drive is at the mid-range of the curve than at levels of activation close to maximum [15]. Therefore, the failure to observe a systematic effect of pain on voluntary activation in previous studies is likely to be explained by a predominance of individuals with high baseline levels of voluntary activation, and thus reduced potential for small changes in voluntary drive (i.e. during pain) to reduce the superimposed twitch response. In previous studies, voluntary activation of over 90% has been commonly reported during maximal isometric knee extensions at 90° knee flexion [23,39]. As the knee extends, there is an almost linear reduction in maximal voluntary activation, and average values lower than 80% have been reported at 60° [23], consistent with the values observed in the present study (non-normalized average: 76%). This reduction in voluntary activation is thought to be associated with reduced facilitation of the central drive from Ia afferents at shorter muscle lengths. The submaximal voluntary activation at 60° of knee extension avoids the non-linearity at the upper end of the sigmoidal force vs. voluntary drive curve and allows for greater resolution in the detection of changes during pain. In other words, the lower levels of voluntary activation reported at this knee angle reduces the ceiling effect that would be observed near 100% voluntary drive curve.

However, given that only a moderate correlation was observed between baseline voluntary activation and the reduction in MVC force (r =—0.62), other factors are likely to contribute to the heterogeneity in pain response. First, as saline-induced pain causes an overestimation of perceived effort [40], participants may have sensed the painful contraction as maximal, despite generation of submaximal force output. This effect has been attributed to reduced cortical excitability, further supporting a reduced voluntary drive. Second, the amplitude of single motor unit twitch force is increased during pain [21]. This increase is believed to involve activation of β_2_ adrenergic receptors in response to noxious stimuli, which modulates the calcium release/re-uptake processes [41]. The faster calcium re-uptake results in increased amplitude and reduced duration of the twitch force, thus reducing the total motor unit force due to reduced fusion of twitches. Last, adaptations to pain can include mechanisms “upstream” of the motor cortex, e.g. at a cognitive level through fear-avoidance mechanisms [42], i.e. at the level of motor planning, although this seems unlikely in the present study (see *Fear and anxiety* below).

The presence of a gradual decrease in resting twitch and MVC force over time indicates that, despite the long rest periods provided, participants developed some muscle fatigue during the experiment [13]. However, as these effects were similar for both hypertonic and isotonic saline injections, muscle fatigue cannot explain the reduced MVC force observed only with hypertonic saline.

### Force direction

In the current study, a three-dimensional force sensor was used to assess the potential changes in total force generated during maximal knee extensions. The resultant force was not perfectly aligned with the sagittal plane, but at 8.7 degrees lateral, on average. Contrary to our third hypothesis, and to what has been previously observed during submaximal contractions [20], there was no systematic change in force angle during pain. The magnitude of change in total force was strongly correlated with changes in the knee extension force component (Fz, r = 0.99). In contrast, only a moderate correlation was found between total force and the media-lateral component (Fx, r = 0.52). Although no significant changes were identified in the activity of auxiliary muscles with pain, non-systematic changes in pelvis position and body posture may have contributed to this lower correlation.

### Fear and anxiety

Fear of movement and psychological distress have been associated with reduced MVC force and voluntary muscle activation in patients with chronic neck and low back pain [10,43]. Contrary to our prediction, we found no relationship between the reduction in MVC force during pain and measures of pain beliefs or anxiety. This disparity between clinical patients and healthy individuals is likely to be attributed to differences in the source of pain and the cognitive responses to it. In chronic conditions, pain represents a real or potential threat (e.g. injury), and inactivity due to fear of pain/re-injury can lead to muscle atrophy and reduced cross-sectional area [8,44,45], directly affecting muscle force. In contrast, injection of hypertonic saline elicits pain for a short period (~10 minutes) with no long-term consequences [46]. Thus the role of fear and anxiety in task performance is less well-defined than in clinical settings. Participants reported anxiety levels from zero to 7.9 cm on the 10-cm VAS, which would imply mild anxiety for most, but high levels for some. However, the broad differences in the reduction of MVC force during pain could not be explained by variations in pain or anxiety levels. It cannot be excluded that participants with extreme pain beliefs might show marked differences in performance during acute experimental pain, but this hypothesis remains to be tested.

The positive correlation between pain and anxiety scores concurs with previous studies on experimental muscle pain [47] and data for patients with rheumatoid arthritis [48] and chronic back and neck pain [49]. Taken together these results demonstrate that unhealthy pain beliefs negatively affect pain perception (e.g. higher pain scores) both in patients and healthy participants [50].

## Conclusion

Experimental knee pain significantly reduced MVC force, but this effect was not systematically observed in all participants. Inter-subject variation in response to pain was largely explained by differences in baseline voluntary activation. Participants with reduced MVC force during pain also presented lower activity of agonist and antagonist muscles, most likely caused by a generalized reduction in the voluntary drive. These results suggest that deficits in voluntary drive are responsible for the reduction in maximal force during pain. Anxiety towards pain exacerbated the cognition of pain intensity.

## Supporting Information

S1 File Experimental data. This files contains all the parameters used for data analysis: Maximal force amplitude, force angle, amplitude of the superimposed and resting twitch responses, voluntary activation, RMS EMG of RF, VM, and VL muscles (ipsilateral side), RMS EMG of GM, ES, and BF muscles (bilateral), pain and anxiety scores, as well as the individual scores of each of the questionnaires assessed.
(XLSX)
